# Constitutional indocyanine green excretion defect in a Chinese patient without underlying liver disease: case report and mechanistic insights

**DOI:** 10.3389/fmed.2026.1701264

**Published:** 2026-02-09

**Authors:** Xueying Ma, Fei Tian, Shuanghao Zhou, Jianjun Li, Yankun Liu, Ying Hui, Xiangming Ma

**Affiliations:** 1 Department of Hepatobiliary Surgery, Kailuan General Hospital Affiliated to North China University of Science and Technology, Tangshan, China; 2 Department of Tumor Chemoradiotherapy, Affiliated Hospital of North China University of Science and Technology, Tangshan, China; 3 Department of Medical Molecular Diagnosis, Tangshan People’s Hospital, Tangshan, China; 4 Tangshan Key Laboratory of Precision Medicine Testing, Tangshan People’s Hospital, Tangshan, China; 5 MRI Division, Department of Imaging, Kailuan General Hospital Affiliated to North China University of Science and Technology, Tangshan, China

**Keywords:** case report, constitutional ICG excretion defect, Gd-EOB-DTPA-enhanced MRI, indocyanine green, whole-exome sequencing

## Abstract

Constitutional indocyanine green excretion defect (CIED) is a rare disorder characterized by markedly delayed plasma indocyanine green disappearance (ICG-R15 > 50%) despite normal results from conventional liver function tests. We present case a case involving 60-year-old woman diagnosed with CIED, which represents the first documented case in a Chinese patient without any underlying liver disease. This case expands the recognized geographic and epidemiologic spectrum of CIED. Whole-exome sequencing revealed no deletions or pathogenic variants in genes encoding established ICG transporters [SLCO1B1 (OATP1B1), SLCO1B3 (OATP1B3), SLC10A1 (NTCP), and ABCC2 (MRP2)], excluding conventional mechanistic explanations. Our integrated clinical and molecular characterization offers novel insights into this rare disorder. These findings suggest a novel pathogenesis independent of known transporter deficiencies, highlighting the need for further research and underscoring the importance of integrating multidimensional clinical data for accurate interpretation of ICG test results.

## 1 Introduction

Constitutional indocyanine green excretion defect (CIED) is a rare disorder characterized by a markedly delayed plasma indocyanine green 15-min retention rate (ICG-R15 > 50%) without any signs of jaundice or other biochemical liver dysfunction (1, 2). Due to its rarity, the specific imaging features associated with condition remain undefined (1). The estimated prevalence in Japan is approximately 0.007% (1). Reported cases of CIED demonstrate a distinct pattern of geographical clustering. A systematic search across the PubMed, Embase, Web of Science, LILACS, and SciELO databases, supplemented by citation tracing of seminal literature, revealed that all cases meeting the stringent diagnostic criteria for CIED are concentrated in East Asia, with the vast majority originating from Japan and only a single documented case reported from China. To date, no confirmed cases of CIED have been identified outside East Asia or among non-East Asian populations. A comprehensive summary of all reported CIED cases is presented in Table 1 (2–12). This distribution pattern likely stems from the routine use of ICG clearance testing during the preoperative evaluation of hepatic malignancies in Japan, in contrast to its more limited application in other regions. Furthermore, the absence of prominent clinical manifestations often leads to its incidental discovery during preoperative assessments.

**Table 1:** Previously reported cases of constitutional indocyanine green excretory defect.

Author	Year	Age/sex	ICG-R15	Disease	Preoperative liver functional evaluation	Nationality
Ikejima et al. (3)	1993	51/M	70	Hepatitis B virus carrier	Liver biopsy	Japan
Hanazaki et al. (4)	2000	47/F	59.8	HCH	GSA liver scintigraphy	Japan
Yamanaka et al. (5)	2001	61/M	72	HCC	GSA liver scintigraphy, liver biopsy	Japan
Kadono et al. (6)	2006	78/F	79.3	IBC	GSA liver scintigraphy, AKBR	Japan
Maeda et al. (7)	2007	69/F	83.8	HCC	BTR	Japan
Aoki et al. (8)	2013	77/M	77.1	HCC	GSA liver scintigraphy	Japan
Kagawa et al. (9)	2017	44/F	90.7	PBC(stage I)	Liver biopsy	Japan
		58/F	79.3	PBC(stage I)	Liver biopsy	Japan
		62/M	86	Fatty liver	Liver biopsy	Japan
		53/F	70.8	HCC	Liver biopsy	Japan
Nakatake et al. (10)	2018	83/M	76.2	HCC	GSA liver scintigraphy	Japan
Liu et al. (11)	2021	68/M	82.9, 84.9	HCC	(Gd-EOB-DTPA)–enhanced MRI	China
Tanimoto et al. (12)	2024	64.5 (32–76)/M (*n* = 3), F (*n* = 3)	74.7 (41.6–84.6)	HCC (*n* = 3) CRLM (*n* = 3)	GSA liver scintigraphy, liver biopsy	Japan
Morikawa et al. (2)	2024	64/M	70	HCC	GSA liver scintigraphy	Japan

ICG-R15, indocyanine green retention rate at 15 minutes; F, female; M, male; HCH, hepatic cavernous hemangioma; HCC, hepatocellular carcinoma; IBC, intrahepatic biliary cystadenoma; PBC, primary biliary cholangitis; CRLM, colorectal liver metastasis; GSA, 99mTc-galactosyl human serum albumin; AKBR, arterial ketone body ratio; BTR, branched chain amino acid and tyrosine ratio; Gd-EOB-DTPA, Gadolinium-ethoxybenzyl-diethylenetriamine pentaacetic acid.

This diagnostic landscape has also led early studies to frequently associate CIED with concurrent liver diseases, such as hepatocellular carcinoma (HCC) or hepatic cavernous hemangioma (HCH) (2–12). Some studies even consider these associations as a consequence of hepatic dysfunction. However, multiple case reports (Table 1) have revealed no evidence of cirrhosis, portal hypertension, or severe active hepatitis on imaging or histological examination in these patients, indicating that CIED is a distinct clinical entity independent of traditional liver diseases. The present case, which demonstrated significant ICG retention (ICG-R15 = 69.1 and 71.1%) without a typical liver disease background, further supports this view and provides a new basis for identifying and diagnosing CIED in the Chinese population.

The kinetics of ICG involve its intravenous injection, followed by specific uptake by hepatocytes and subsequent excretion unchanged into the bile, ultimately leading to its elimination via the intestines (13). The uptake process is primarily mediated by organic anion transporting polypeptide 1B3 (OATP1B3, encoded by the SLCO1B3 gene), located on the sinusoidal membrane of hepatocytes (14–16), which exhibits nanomolar-level high affinity for ICG (17); OATP1B1 (SLCO1B1) from the same family may also play an auxiliary role (1, 15). The ICG-R15 is a core indicator for assessing functional hepatic reserve, defined specifically as the percentage of ICG retained in the blood 15 min after injection. A lower ICG-R15 value typically reflects superior hepatic blood flow perfusion and hepatocellular function; an ICG-R15 exceeding 10% suggests the onset of impaired liver function and decreased hepatic reserve, while a value greater than 50% signifies severely compromised excretory capacity and substantial hepatic dysfunction. In this case, the two ICG-R15 measurements were 69.1 and 71.1%, indicating markedly delayed ICG excretion; however, all other liver function parameters remained within normal limits, leading to its definition as CIED.

The current pathophysiological understanding attributes CIED to defects in the hepatocellular basolateral OATP transporters (1, 16). The core mechanism lies in the downregulation of OATP1B3 expression or function, which directly results in a severe reduction in the efficiency of ICG entry from the portal vein and hepatic arterial blood into the sinusoidal membrane of hepatocytes, thereby manifesting as an elevated level of ICG-R15 (14–16). Given that the hepatocellular uptake of gadolinium-ethoxybenzyl-diethylenetriaminepentaacetic (Gd-EOB-DTPA) is also highly dependent on OATP1B3 (and OATP1B1), this defect is expected to similarly lead to reduced liver parenchymal enhancement in the hepatobiliary phase of magnetic resonance imaging (MRI) (1). However, this reported case of CIED, investigated via Gd-EOB-DTPA-enhanced MRI and whole-exome sequencing—which revealed no deletions or pathogenic variants in the genes encoding these OATP transporters—challenges this paradigm and suggests novel mechanistic insights.

### 1.1 Case description

The patient was a 60-year-old woman scheduled for laparoscopic cholecystectomy due to gallbladder stones. Preoperative evaluation revealed abnormal ICG metabolism, with an ICG-R15 of 69.1%. Her personal and family history was unremarkable: she denied alcohol consumption, smoking, substance abuse, or any history of hepatobiliary disease, and there was no family history of liver or hereditary disorders. Physical examination revealed no positive signs. Laboratory tests showed a total bilirubin level of 19.3 μmol/L, with albumin, coagulation profile, and liver enzyme levels all within normal limits. Her Child-Pugh score was 5 (Class A). Additionally, transient elastography demonstrated both liver stiffness measurement (LSM) (5.5 kPa) and controlled attenuation parameter (CAP) (237 dB/m) within normal ranges, ruling out liver fibrosis or hepatic steatosis.

**Figure 1:**
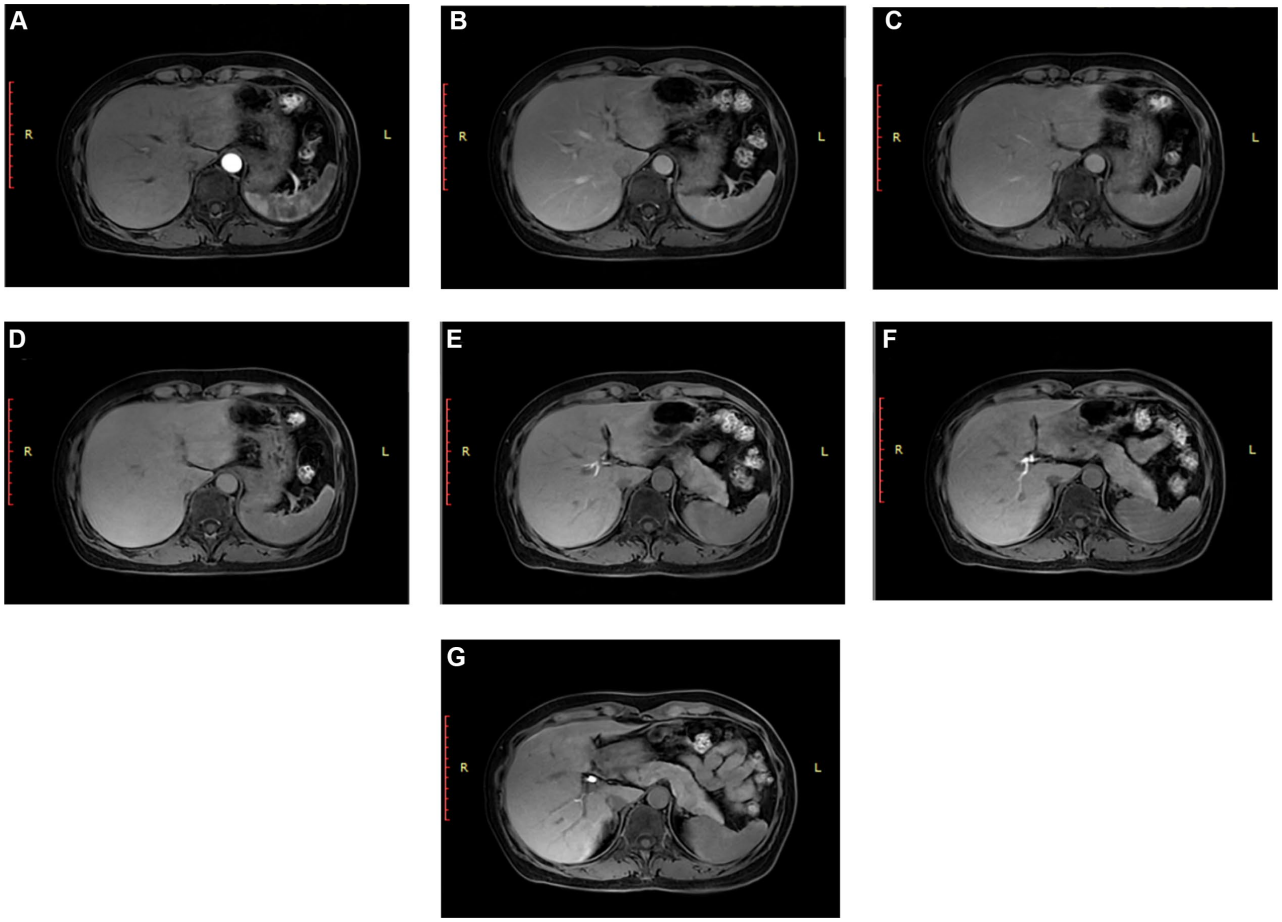
Gd-EOB-DTPA-enhanced hepatobiliary MRI. **(A)** Arterial phase, **(B)** portal venous phase, **(C)** delayed phase; **(D)** transitional phase, **(E)** hepatobiliary phase (20-min post-contrast), **(F)** hepatobiliary phase (25-min post-contrast), and **(G)** hepatobiliary phase (30-min post-contrast).

To exclude potential confounding effects of gallbladder pathology on ICG kinetics, repeat ICG clearance testing was performed 3 months postoperatively with the patient’s signed informed consent. The result showed an ICG-R15 of 71.1%. Liver function tests—including bilirubin, albumin, and coagulation parameters—remained within normal limits. Given the paradoxical findings of abnormal ICG kinetics in the context of otherwise normal liver function, a preliminary diagnosis of CIED was considered. To further clarify the etiology, Gd-EOB-DTPA-enhanced MRI was performed to accurately assess the hepatic function and structure. The imaging procedure and findings were as follows (Figure 1): After intravenous injection of gadoxetate disodium, dynamic scans were acquired at the following phases: Arterial phase (Figure 1A): Complete enhancement of the hepatic artery, indicating normal arterial supply. Portal venous phase (Figure 1B): Enhancement observed in both the hepatic and portal veins, with peak parenchymal enhancement. Delayed phase (Figure 1C): Parenchymal and vascular enhancement diminished appropriately, consistent with normal contrast metabolism. Transitional phase (Figure 1D): Homogeneous signal intensity in liver parenchyma and intrahepatic vessels, with no focal defects or abnormal perfusion. Hepatobiliary phase (20-, 25-, and 30-min post-injection) (Figures 1E–G): Sequential opacification of the biliary tree and contrast excretion into the common bile duct, indicating intact and functional hepatocellular uptake and biliary excretion pathways.

Gd-EOB-DTPA-enhanced MRI revealed no abnormalities in the hepatocellular uptake, intracellular processing, or biliary excretion of Gd-EOB-DTPA, indicating preserved hepatic structure and function. This phenotypic profile closely aligns with a previously reported case of CIED in China from 2021, which also demonstrated markedly elevated ICG-R15 (82.9 and 84.9%) in the setting of normal Gd-EOB-DTPA-enhanced MRI and conventional liver function tests. Given that this contrast agent shares key hepatocyte membrane transporters with ICG—including OATP1B1/B3 and NTCP during the uptake phase and MRP2 during the excretion phase (18) —the imaging findings provide compelling evidence that these transporter-mediated pathways remain structurally and functionally intact in this patient. Consequently, Gd-EOB-DTPA-enhanced MRI helped exclude ICG retention due to dysfunction of these transporters, further supporting the diagnosis of CIED and pointing toward etiology potentially related to ICG-specific, yet unidentified, metabolic or excretory processes. In this case, Gd-EOB-DTPA-enhanced MRI played a pivotal role in the differential diagnosis. The observation of normal MRI findings in the context of a markedly elevated ICG-R15 served as a key indicator for distinguishing CIED from parenchymal liver disease.

To investigate molecular pathogenesis, whole-exome sequencing (WES) was performed with the patient’s written informed consent. The analysis specifically targeted genes encoding established ICG transporters—including SLCO1B1 (OATP1B1), SLCO1B3 (OATP1B3), SLC10A1 (NTCP), and ABCC2 (MRP2) (16) —yet no pathogenic variants were identified. This finding effectively excludes defects in these transporter-encoding genes as the underlying mechanism of CIED in this case. Instead, it suggests that the condition may arise from alterations in other, as-yet unidentified regulatory genes or pathways. Consequently, we extended our investigation to analyze additional variants detected through WES to explore potential genetic contributors to CIED beyond the canonical transporter machinery. Interaction network analysis (BioGRID, STRING, IntAct, GeneMANIA, KEGG) and literature retrieval explored potential links to ICG transport pathways or the hepatic microenvironment. The proposed mechanisms are summarized in Supplementary Table S1.

## 2 Materials and methods

### 2.1 Data collection and clinical evaluation

Clinical data were collected from medical records, including demographic information, medical history, physical examination findings, and laboratory results. Serum biochemical parameters—such as liver function tests, coagulation profiles, and bilirubin levels—were measured using standard automated clinical laboratory analyzers to assess hepatic function. LSM and CAP were evaluated using transient elastography (FibroTouch FT1000, Wuxi Hisky Medical Technology, China). Examinations were performed by trained operators following the manufacturer’s protocol, with the patient in the supine position and the right arm maximally abducted. A total of 10 valid measurements were obtained, and the median LSM (expressed in kPa) and median CAP (expressed in dB/m) were recorded to assess liver fibrosis and steatosis.

### 2.2 Indocyanine green clearance testing

Indocyanine green clearance testing was performed before laparoscopic cholecystectomy and again 3 months postoperatively. Indocyanine green for injection (Dandong Yichuang Pharmaceutical Co., Ltd., China) was reconstituted with the supplied sterile water for injection to a concentration of 5 mg/mL. A dose of 0.5 mg/kg body weight was administered as an intravenous bolus for 10 s. Blood ICG concentration was monitored non-invasively and continuously using an ICG clearance meter (Model A; Shinsei Electric Co., Ltd., Japan) with a finger-clip optical sensor. The ICG-R15 was automatically calculated by the device’s built-in software. Testing was conducted with the patient in a fasting state, following an overnight fast. The patient was at rest, and positioned supine during the procedure.

### 2.3 Gd-EOB-DTPA-enhanced magnetic resonance imaging

MRI examination was performed using a uMR OMEGA 3.0-T scanner (United Imaging Healthcare Co., Ltd., China) to evaluate hepatic parenchymal enhancement, biliary excretion, and vascular architecture. Following intravenous injection of gadoxetate disodium (Bayer HealthCare Pharmaceuticals, Germany) (dose: 0.025 mmol/kg; injection rate: 1.0 mL/s), dynamic imaging was acquired during the following phases: arterial phase, portal venous phase, delayed phase, transitional phase, and hepatobiliary phase (20-, 25-, and 30-min post-injection). Image analysis was performed independently by two experienced radiologists blinded to the ICG-R15 results.

### 2.4 Whole-exome sequencing and bioinformatics analysis

Whole-exome sequencing was performed on the proband using capture-based next-generation sequencing technology. Genomic DNA was extracted, libraries were constructed, and the exonic regions were captured and enriched using a hybridization probe panel. Paired-end sequencing was completed on an Illumina NovaSeq series or BGI T7 sequencing platform. The mean sequencing depth was 131×, with 99.73% of target bases covered and 98.56% covered at ≥20×. The reference genome was GRCh37/hg19.

The bioinformatics analysis pipeline proceeded as follows: raw sequencing data were demultiplexed based on library barcodes and converted from bcl to fastq format; adapter sequences and low-quality/overly short reads were trimmed using cutadapt; reads were aligned to the hg19 reference genome using BWA-MEM. GATK was used for base quality score recalibration and local realignment around indels to enhance variant-calling accuracy. Subsequently, detection and annotation of single-nucleotide variants and small insertions/deletions were performed, integrating multiple disease and phenotype databases (e.g., ClinVar, OMIM, HGMD, and HPO), population frequency databases (e.g., gnomAD, ExAC, and 1,000 Genomes), and *in silico* prediction tools (e.g., SIFT, PolyPhen-2, CADD, and REVEL) for comprehensive assessment.

Variant filtering and prioritization were performed in conjunction with the clinical phenotype, using phenotype-correlation tools to score variants, and the process involved the following steps: removal of variants with excessively high population allele frequencies (>2%), low sequencing depth, or abnormal variant allele fractions; exclusion of polymorphic sites present in in-house normal controls and synonymous variants unreported in databases; and retention of previously reported pathogenic variants. The Final medical interpretation was based on the match between genotype and phenotype, inheritance patterns, population frequency, and other relevant information, with pathogenicity classification conducted according to the standards and guidelines of the American College of Medical Genetics and Genomics (ACMG). Candidate variants identified by next-generation sequencing were validated using Sanger sequencing; if copy number variations were detected, quantitative real-time PCR was employed for confirmation.

### 2.5 Interaction network and pathway analysis

Multiple bioinformatics tools and databases were utilized for protein–protein interaction network analysis, including GeneMANIA, STRING, and BioGRID. Pathway enrichment analysis was performed based on the Kyoto Encyclopedia of Genes and Genomes (KEGG) database. Literature mining was conducted to identify potential mechanistic links between candidate genes and pathways involved in hepatic transporter regulation, growth hormone signaling, bile acid metabolism, or inflammatory responses.

## 3 Discussion

CIED remains exceptionally rare, with incompletely elucidated pathogenesis. Previous immunohistochemical and RT-PCR analyses of liver tissue from ICG excretion defect patients identified absent or minimal OATP1B1/B3 expression, supporting basolateral OATP deficiency as the principal defect (1, 16). Notably, these studies were predominantly conducted in patients with HCC, and HCC itself can lead to the downregulation of OATP1B1/B3 expression (1, 16).

This case challenges the conventional OATP1B1/B3 deficiency hypothesis based on four critical observations: (1) Absence of liver disease and normal bilirubin handling: No chronic liver disease history; persistently normal bilirubin metabolism and Child-Pugh A status that excluded cholestasis. Bilirubin uses the same OATP1B1/B3 (uptake) and MRP2 (efflux) pathways as ICG and Gd-EOB-DTPA (18). Bilirubin kinetics are normal, implying that global transporter function is retained. (2) Normal Gd-EOB-DTPA-enhanced MRI kinetics: Gd-EOB-DTPA utilizes the same uptake (OATP1B1/B3 and NTCP) and efflux (MRP2) pathways (18). Normal hepatobiliary phase enhancement confirms the functional integrity of these transporters. (3) Persistent ICG-R15 elevation pre−/post-cholecystectomy: Markedly elevated ICG-R15 (69.1% pre-op; 71.1% post-op) excluded gallbladder pathology as a confounder. (4) No pathogenic variants in ICG transporter genes: Comprehensive whole-exome sequencing revealed no deleterious variants in SLCO1B1, SLCO1B3, SLC10A1, or ABCC2. The absence of relevant pharmacotherapy excluded acquired OATP inhibition (19, 20). We conclude that CIED in this patient is not due to defects in known ICG transporter genes, indicating the involvement of hitherto unrecognized pathogenic mechanisms.

In clinical practice, CIED should be considered in patients with markedly prolonged ICG-R15 after excluding parenchymal liver disease and cholestasis. Clinicians should integrate conventional liver function tests, imaging (e.g., Gd-EOB-DTPA-enhanced MRI), and potentially genetic analysis for a comprehensive assessment to avoid misdiagnosis of reduced functional hepatic reserve. Gd-EOB-DTPA-enhanced MRI shows promise in evaluating hepatic transporter function and may emerge as a novel method of quantitative liver reserve assessment. Although no OATP1B1/B3 defects were identified here, patients with confirmed or suspected CIED should remain vigilant for potential impaired uptake and associated toxicity risks of drugs reliant on OATP1B1/B3 function (16). Individualized pharmacological assessment may be warranted.

We report the first Chinese case of CIED without an underlying liver disease. Systematic assessment—serial ICG clearance testing, Gd-EOB-DTPA-enhanced MRI, and whole-exome sequencing—excluded conventional mechanisms involving deficient OATP1B1/B3 or other known transporters. We propose four potential mechanistic hypotheses to explain the observed phenotype:

1. Dysregulation of growth hormone (GH) signaling: growth hormone (GH) binds specifically to the growth hormone receptor (GHR) on the hepatocyte membrane, inducing the dimerization of two GHR molecules. This activates the associated JAK2 kinase, which phosphorylates both GHR and itself. Signal transducer and activator of transcription 5 (STAT5) is then recruited to the phosphorylated GHR and is itself tyrosine-phosphorylated by JAK2. The phosphorylated STAT5 (particularly STAT5b) dissociates from the receptor, forms dimers, and translocates into the nucleus as a signaling messenger. Within the nucleus, the activated STAT5 dimer binds directly to a specific site in the OATP1B3 promoter (positions −170 to −162; sequence 5′-TTCTGAGAA-3′). Similarly, STAT5 binding sites are present in the promoter region of the NTCP gene. This binding acts as a “key” that turns on the transcriptional “switch,” significantly enhancing the transcriptional activity of both OATP1B3 and NTCP genes. Increased transcription elevates the messenger RNA (mRNA) levels of OATP1B3 and NTCP. These mRNAs exit the nucleus and serve as templates in the cytoplasm, guiding ribosomes to synthesize increased amounts of OATP1B3 (approximately 2.1-fold) and NTCP proteins (21–23).

The activated STAT5 dimer in the nucleus also binds to specific regions of the HNF6 gene promoter. This binding initiates and significantly enhances HNF6 transcription, resulting in a substantial increase in HNF6 mRNA and protein levels within the nucleus. The HNF6 protein then binds to the promoters of downstream transcription factors, including Foxa2, HNF1α, and HNF4α, potently upregulating their expression. This results in a sharp rise in the nuclear concentrations of Foxa2, HNF1α, and HNF4α. Through this two-stage amplification, the initial GH signal is transformed into a marked increase in the levels of multiple potent transcription factors. Subsequently, the elevated concentrations of Foxa2, HNF1α, and HNF4α proteins act as “executors,” cooperatively binding to the promoter and enhancer regions of transporter genes such as MRP2, NTCP, and OATP1B1. This robustly initiates the transcription of these genes, and the resulting mRNAs are translated into functional MRP2, NTCP, and OATP1 proteins, which are integrated into the cell membrane to perform their transport functions (24, 25).

Additionally, the activated STAT5 dimer in the nucleus collaborates with the transcription factor NF-Y. NF-Y first binds to the MRP2 gene promoter, altering the chromatin structure to an “open” and accessible state. STAT5 then binds to this NF-Y–primed DNA region, cooperatively initiating MRP2 gene transcription. The transcribed MRP2 mRNA is transported to the cytoplasm, where it serves as a template for the synthesis of abundant MRP2 protein (25).

Together, these mechanisms constitute a multi-tiered network through which GH regulates hepatic transporters. Given that ICG—a dye commonly used in liver function tests—relies on the function of hepatocyte membrane transporters for its excretion, we hypothesize that the GH signaling pathway may influence ICG uptake and excretion by modulating the expression of these transporters. In this case, the identified GH1 (variants of uncertain significance) (c.615C > G, p.Ile205Met) may interfere with GH-GHR binding, leading to abnormal GHR dimerization or conformational changes. Alternatively, it could disrupt GH stability or secretion, impairing folding, secretion, or half-life, thereby reducing effective circulating GH levels and diminishing JAK2-STAT5 activation. This cascade ultimately downregulates OATP1B3 expression, contributing to impaired ICG uptake. GH1 signaling defects may represent a novel category of “acquired ICG excretion dysfunction.” We recommend screening for the GH axis in patients with unexplained ICG retention and avoiding substrates of MRP2, OATP1B1, OATP1B3, and NTCP [e.g., statins metabolized via OATP1B3 (15)]. Other genes, such as TLR3 (2) and IL10RA (13), may interfere with the GH-GHR-JAK2-STAT5 signaling cascade, thereby indirectly impairing transporter activity and resulting in defective ICG uptake.

Compared to previous studies on CIED, the present case for the first time establishes a potential link between dysregulation of the GH1 signaling pathway and impaired ICG excretion, thereby expanding the pathophysiological understanding of this disorder. While previous studies have predominantly focused on mutations or functional defects in the transporters themselves, our findings suggest that dysregulation of upstream hormonal signaling pathways may similarly lead to a phenotypically comparable defect in hepatic excretory function. This insight offers a novel perspective for elucidating the molecular mechanisms underlying CIED.

1. Hepatocellular microenvironment disruption: a disordered hepatocyte metabolic microenvironment impedes ICG transport. Genetic defects affecting hepatic lipid metabolism (26–32), signal dysregulation (29, 31, 33–36), inflammatory responses (26, 27, 31, 33, 34), and fibrogenic processes (26–28, 31, 33–35) may indirectly suppress ICG transporter function through microenvironmental alterations.
2. Hepatocyte–biliary coordination defects: the hepatocyte–biliary coordination impairment hypothesis proposes that variants in biliary development genes (27, 31, 37–39) may cause biliary dysfunction, potentially reducing hepatocyte ICG excretion capacity.
3. Polygenic additive effects: multi-gene synergistic effects result in “borderline functional impairment.”

These findings provide novel directions for elucidating CIED pathogenesis.

The clinical implications of this study are profound and far-reaching. It directly addresses the current diagnostic challenges in clinical practice for patients with unexplained ICG retention, providing critical pathophysiological insights and precise solutions for this ambiguous clinical scenario. The findings strongly caution that reliance solely on the indocyanine green retention test (ICG-R15) may lead to significant misjudgment of hepatic function, potentially even inappropriately depriving eligible patients of opportunities for curative surgery. Consequently, we explicitly propose a transformative diagnostic pathway: for patients with abnormally elevated ICG-R15, functional validation using Gd-EOB-DTPA-enhanced MRI should be used in combination. This strategy directly targets the “false-positive” pitfall of ICG testing, effectively identifying individuals with actual normal hepatic functional reserve who exhibit ICG retention due to specific transporter dysfunction. By doing so, it enhances the accuracy and equity of surgical decision-making, possessing the immediate potential to alter clinical practice and improve patient outcomes.

Furthermore, this study systematically elucidates the potential phenotypes of CIED and their underlying molecular mechanisms. This represents not merely a diagnostic paradigm shift but also a crucial alert regarding medication safety. It unequivocally indicates that if the defect involves key transporter proteins such as OATP1B1, OATP1B3, NTCP, or MRP2, clinicians must maintain a high index of suspicion for associated drug safety risks. The pharmacokinetics of numerous medications metabolized via these pathways (e.g., statins, hypoglycemic agents, and anticancer drugs) may be similarly compromised, posing potential risks of reduced efficacy or accumulated toxicity (15, 21). Consequently, these findings provide a critical theoretical foundation for the individualized and precise pharmacotherapy of this patient population, extending the management scope from diagnostic accuracy alone to long-term medication safety monitoring.

We fully acknowledge the prior reports of similar cases from Japan and China, which collectively established that CIED can exist independently of parenchymal liver dysfunction. However, the present case exhibits several critical distinctions from previously reported cases:

1. Novel etiological mechanism: previous cases have primarily focused on functional or expressional abnormalities of hepatocyte membrane transporters, such as OATPs or MRP2. In contrast, this case proposes, for the first time, that a GH1 gene variant may impair ICG excretion by disrupting the JAK2/STAT5 signaling pathway, consequently affecting the expression of downstream transporters. This mechanism introduces a new hormonal regulatory perspective on the etiology of CIED.
2. Distinct clinical context: this case demonstrates persistently elevated ICG-R15 in the absence of typical underlying liver diseases, distinguishing it from most previously reported cases associated with conditions such as HCC, HCH, or fatty liver disease.
3. Expanded ethnic and geographic scope: originating from a non-Japanese population, this case helps challenge the perception that CIED is largely confined to Japanese individuals, suggesting a potentially broader genetic and clinical spectrum for this condition.

In summary, this case not only expands the recognized phenotypic boundaries of CIED but also proposes a novel mechanistic hypothesis, thereby providing a new direction for understanding the regulatory network governing ICG metabolism.

While this study, through systematic clinical and molecular investigation, proposes for the first time a link between dysregulated GH1 signaling and CIED—introducing a novel mechanistic hypothesis beyond conventional transporter defects—several limitations must be acknowledged. First, as a single case report, the findings, while suggestive, render the causal relationship between the identified GH1 variant (c.615C > G, p.Ile205Met) and impaired ICG metabolism speculative, necessitating confirmation through large-scale population screening and functional studies. Second, the proposed molecular pathway—whereby disruption of the GH-JAK2-STAT5 signaling axis downregulates hepatic transporter expression and thereby affects ICG handling—currently relies primarily on bioinformatic analysis and literature inference, without direct mechanistic validation in cellular or animal models. Therefore, we plan to prioritize functional experiments targeting the GH signaling pathway in subsequent research. This will include validating the impact of the identified GH1 variant on STAT5 activation and the expression and function of downstream transporters (OATP1B3, NTCP, MRP2) using *in vitro* hepatocyte models. Concurrently, at the clinical level, systematic screening of the GH axis—encompassing GH1 gene sequencing and serum GH and IGF-1 level measurements—in a larger cohort of patients with unexplained ICG retention will help clarify the prevalence of this pathway dysregulation and its associated clinical phenotypes. These validation efforts are crucial to establishing the generalizability and biological significance of our findings and may ultimately inform future evidence-based precision management strategies.

## 4 Conclusion

Through a comprehensive investigation of a CIED case in a Chinese patient without underlying liver disease, encompassing systematic clinical, imaging, and molecular analyses, this study yields the following conclusions:

First, this case confirms that CIED can exist as a distinct functional defect entity, independent of traditional parenchymal liver disease. This reinforces the necessity of a multidimensional clinical assessment beyond conventional liver function tests when evaluating unexplained ICG retention.

Second, this study provides groundbreaking mechanistic insights. Comprehensive genetic sequencing and functional MRI collectively excluded defects in known transporters—OATP1B1/B3, NTCP, and MRP2—as the direct cause in this patient. Consequently, we propose a novel upstream regulatory hypothesis: a GH1 gene variant may disrupt growth hormone signaling, indirectly leading to dysregulation of the hepatocyte transporter network. This introduces a new “hormone-signaling-function” perspective for understanding CIED and liver transporter physiology.

Third, our findings hold immediate clinical-translational significance. They clearly demonstrate that reliance solely on the ICG-R15 index can lead to severe misjudgment of functional hepatic reserves. We therefore recommend integrating Gd-EOB-DTPA-enhanced MRI as a key diagnostic tool. Furthermore, this study expands the management scope of CIED from diagnostic accuracy to long-term medication safety vigilance. It highlights the need to assess potential risks associated with drugs metabolized by key transporters such as OATPs and MRP2 in these patients, thereby providing a basis for truly individualized pharmacotherapy.

Future research should prioritize functional characterizing the GH1 variant and investigating its prevalence in the population. To advance this research, developing novel diagnostic models that integrate genotype, functional imaging, and clinical phenotype is essential. This approach represents not only the key to unraveling the etiology of certain CIED cases but also the foundation for constructing a next-generation “precision liver function assessment” framework. Ultimately, this may lead to the creation of an integrated diagnostic algorithm and evidence-based management strategies, moving beyond the current era of diagnostic ambiguity and ushering in a new epoch of more precise, safer, and highly personalized liver function evaluation.

## Data Availability

The original contributions presented in the study are included in the article/Supplementary material, further inquiries can be directed to the corresponding author.
