# Supplementary material for: Constitutional indocyanine green excretion defect in a Chinese patient without underlying liver disease: case report and mechanistic insights
Source: Front Med (Lausanne). 2026 Feb 9;13:1701264. doi: 10.3389/fmed.2026.1701264 (PMC12926837; doi:10.3389/fmed.2026.1701264)
Supplement: Supplementary file 1 [file Table_1.docx]

Supplementary Table S1. Potential mechanistic hypotheses of ICG excretion deficiency

| Gene name | ACMG classification | Correlation | Potential mechanism |
| --- | --- | --- | --- |
| GH1 | VUS | +++ | dysregulated GH signaling |
| ITGB4 |  |  |  |
| TLR3 |  |  |  |
| DUOX2 | LP | +++ | hepatocyte-biliary coordination defects |
| CRB1 |  |  |  |
| FGF8 | VUS |  |  |
| LCP2 |  |  |  |
| JAG1 |  |  |  |
| DUOX2 | LP | ++ | hepatocellular microenvironment disruption |
| CPT1A | VUS |  |  |
| CLPB |  |  |  |
| PDE11A |  |  |  |
| SLC1A4 |  |  |  |
| ITGB4 |  |  |  |
| ASAH1 |  |  |  |
| TLR3 |  |  |  |
| IL10RA |  |  |  |
| CBLB |  |  |  |
| JAG1 |  |  |  |
| SAMHD1 |  |  |  |
| DUOX2 | LP | + | polygenic additive effects |
| CRB1 |  |  |  |
| SAMHD1 | VUS |  |  |
| CPT1A |  |  |  |
| CLPB |  |  |  |
| PDE11A |  |  |  |
| SLC1A4 |  |  |  |
| ITGB4 |  |  |  |
| ASAH1 |  |  |  |
| TLR3 |  |  |  |
| IL10RA |  |  |  |
| LCP2 |  |  |  |
| JAG1 |  |  |  |
| GH1 |  |  |  |
| CBLB |  |  |  |
| FGF8 |  |  |  |

Note: ACMG: American College of Medical Genetics and Genomics; LP: Likely Pathogenic; VUS: Variants of Uncertain Significance; +++: Strong association (causality supported by experimental evidence); ++: Moderate association (mechanistically plausible but requiring further validation); +: Weak association (hypothetical, based on co-occurrence or bioinformatic predictions)
